# Economic burden of toxicities associated with treating metastatic melanoma in eight countries

**DOI:** 10.1007/s10198-015-0757-y

**Published:** 2015-12-31

**Authors:** Elizabeth Wehler, Zhongyun Zhao, S. Pinar Bilir, Julie Munakata, Beth Barber

**Affiliations:** 1 IMS Health, 1 IMS Drive, Plymouth Meeting, PA 19462 USA; 2 Amgen, 1 Amgen Center Drive, Thousand Oaks, CA 91320 USA; 3 IMS Health, 425 Market Street, 7th Floor, San Francisco, CA 94105 USA

**Keywords:** Adverse drug event, Melanoma, Costs and cost analysis, Cost of illness, L19

## Abstract

**Background:**

Information on costs of managing adverse events (AEs) associated with current treatments in metastatic melanoma is limited. This study estimates costs of AEs in eight countries: Australia (AU), Canada (CA), France (FR), Germany (GE), Italy (IT), the Netherlands (NL), Spain (ES), and the UK.

**Methods:**

A literature search was conducted to identify grade 3/4 AEs from product label, published trials, conference abstracts, and treatment guidelines. Resource utilization for the management of each type of AE was determined via interviews with 5 melanoma clinicians in each country. Outpatient and inpatient costs were estimated for each type of AE using country-specific tariffs or government/published sources.

**Results:**

In outpatient settings, the most costly AEs per incident included cutaneous squamous cell carcinoma (CSCC) (€1063, £720; NL/UK), anemia (€1443, €1329, €1285; ES/IT/FR), peripheral neuropathy (€1289; ES), and immune-related diarrhea (AUS$1,121; AU). In inpatient settings, the most costly AEs per hospitalization included hypophysitis (€10,265; €5316; CAN$9735; AUS$7231: ES/FR/CA/AU), dyspnea (€9077; GE), elevated liver enzymes (€6913, CAN$8030, AUS$6594; FR/CA/AU), CSCC (CAN$8934; CA), peripheral neuropathy (€6977, €4144, CAN$9472; NL/ES/CA), and diarrhea (£4284, €4113; UK/ES).

**Conclusions:**

Costs of managing AEs can be significant, and thus effective treatments with lower rates of severe AEs would be valuable.

**Electronic supplementary material:**

The online version of this article (doi:10.1007/s10198-015-0757-y) contains supplementary material, which is available to authorized users.

## Background

Melanoma is a globally significant condition, with approximately 200,000 incident melanoma skin cancers and an estimated 46,000 fatalities occurring from advanced forms of the disease in 2008 [1]. About 85 % of these melanoma cases occur in developed countries and represent the sixth most commonly diagnosed cancer (5.6–39.3 per 100,000), with regions such as North America, Europe, and Australia reporting the highest incidence rates in the world [1]. Throughout recent years, the age-standardized incidence rate of melanoma has continued to increase approximately 4–6 % per year in European countries such as the UK, France and Germany [2]. As melanoma continues to impact growing numbers of individuals, the potential treatment options and associated economic consequences will require careful consideration.

Prior to the introduction of newer treatments for metastatic melanoma in 2011, progress in melanoma treatment had been slow and survival rates had essentially been unchanged for decades. Before the introduction of the newer agents, nearly all patients with regional or distant metastases were treated with on-label and off-label older conventional agents as monotherapy or in combinations and regimens. Three of the most commonly used older conventional agents are dacarbazine (DTIC), fotemustine (only in some countries in Europe) and interleukin-2 (IL-2) (only in the US) [3, 4]. Notably, none of these treatments has demonstrated a clinically meaningful improvement in overall survival in a randomized controlled trial (RCT), and none of these options has been shown to be a superior option [5].

In recent years, significant progress has been made in the fight against metastatic melanoma. Since 2011, six agents have been approved for the treatment of advanced melanoma: ipilimumab, a CTLA-4 antibody; two BRAF inhibitors, vemurafenib and dabrafenib, a MEK inhibitor, trametinib, available for patients with BRAF-mutant melanoma, which occurs in 40–50 % of patients with melanoma; pembrolizumab and nivolumab, PD-L1 inhibitors, were approved in the second half of 2014, and were not yet available at the time this study was conducted [6, 7].

While chemotherapy and IL-2 treatments are most likely to lead to hematologic toxicities such as neutropenia or anemia, BRAF inhibitor studies show greater rates of squamous cell carcinomas and/or keratoacanthoma, and grade 3 or 4 adverse events with the MEK inhibitor treatment include hypertension and rash. Treatment with ipilimumab is associated with a number of grade 3 and 4 adverse events, the majority of which are immune-related, which may involve the gastrointestinal, liver, skin, nervous, endocrine, ocular, or other organ systems [8].

Although trial data demonstrate the extent of AEs experienced on each course of therapy, the economics of managing AEs around the world is not well understood, especially given the different AE profiles from newer therapies. Therefore, this study explores the costs in Italy, Spain, Germany, France, the Netherlands, the UK, Canada, and Australia related to managing the more frequent therapy-related toxicities to better understand the burden of AE-related economic impact.

## Methods

This study estimates payer perspective costs associated management of AEs occurring while patients receive treatment with monotherapy agents that are approved for use and/or referenced in treatment guidelines for first or second line treatment of metastatic melanoma in any of the included countries [9–14]. Products include dacarbazine or DTIC, temozolomide, fotemustine, IL-2, ipilimumab, vemurafenib, dabrafenib, and trametinib. The approach included three steps: (1) identification of toxicities and their rates; (2) detailed interviews with five clinical experts per country to determine a potential range of resource utilization for AE management per setting; and (3) identifying unit costs per resource and calculating total AE costs in each country from a public payer perspective.

### AE identification

Appropriate clinical studies from which to identify a list of severe (grade 3 or 4) adverse events associated with the treatment options of interest were collected via a literature search. Searches were conducted in PubMed, and additional information was provided by publicly available sources from relevant professional conferences, and materials referenced in product prescribing information. Search terms included the drug name (dabrafenib, dacarbazine or DTIC, fotemustine, ipilimumab, interleukin-2, temozolomide, trametinib and vemurafenib) along with “melanoma” and “metastases” or “metastatic” or “advanced”, and “clinical trial”. In order to ensure the study captured all relevant AEs across considered therapies, the highest-quality study was selected for each therapy by line of treatment (first line, second line, or in cases where no second line option was available, mixed line). Study selection criteria prioritized phase 3 studies (unless no phase 3 studies were available), large sample size, and the use of a guideline-recommended dosing regimen. This was done to ensure that AEs were treatment-related, which may be less clear in earlier-phase studies. Severe adverse events, grade 3 and 4 as defined by the National Cancer Institute (NCI) Common Terminology Criteria for Adverse Events (CTCAE), were identified in the selected studies. Any AEs reported in less than 1 % of patients across all selected studies were excluded from the analysis, and additional abnormal lab value AEs were excluded following the first two clinical expert interviews upon indication that no active management would occur. Resource utilization responses during these first two interviews also supported grouping AEs by general treatment approach or degree of management required for later costing, although this did not affect the interviews. An interview guide containing the final list of AEs was compiled by type of therapy to allow clinicians to provide resource utilization answers according to AE etiology where relevant.

### Resource utilization pattern identification

A total of five melanoma clinical experts were interviewed in each country to document their professional management of each AE. Initially, experts were approached if they had recent publications in the field of advanced/metastatic melanoma, melanoma society or conference participation; some participants were identified with the assistance of recruiting services. Physicians were required to treat at least 5 cases of advanced/metastatic melanoma per month; this number was chosen to allow participation in countries with lower melanoma treatment density (e.g. Italy, Canada), although 10–20 cases per month were preferred in other countries (e.g. Australia, Germany). The individuals were experienced in the fields of medical oncology or dermatology.

For each grade 3 or 4 AE per therapy type in the interview guide, physicians were asked to provide details for how they would treat each event. The interview guide was arranged by therapy type, with a list of drugs for which each adverse event occurred to allow physicians to note whether AE treatment would differ by melanoma therapy. Specific questions were translated into local language in Germany, Italy, and Spain, capturing the English-language questions of “what percentage of the time would you hospitalize a patient with this AE,” “for how long would patients be hospitalized” and “for those patients not hospitalized, what outpatient resources would you use: what kinds of tests, procedures or drugs would you recommend, and at what doses or frequencies?” All other interviews were conducted in English.

Expert responses from the five physicians in each country were collated to determine the base case approach. Given an AE without a definitive majority (at least three out of five physicians in agreement), agreement between two out of five physicians was used as the default, if the other physician responses differed. Resources with different names but the same indication were considered to constitute physician agreement (e.g. “oral steroid therapy” or “oral anti-emetic therapy” regardless of specific drug name). Sensitivity analysis calculations were conducted by using any additional or different resources mentioned by clinical experts. In some settings, physicians were reluctant to provide resource use for adverse events they never see in their patients. This was likely because in clinical practice, physicians managed AEs at a lower grade, prior to grade 3 or 4 severity. Results note where this was the only response available.

### Unit costing

Unit costs for required resources identified by clinical experts were collected according to a public payer perspective. All costs were obtained in 2013 local currency and are inflated to 2014 local currency [15–17]. For completeness of data collection, all inpatient costs were recorded even if physicians unanimously agreed that the patient would not be hospitalized for that AE. A diagnosis-related group (DRG) encompassing the relevant condition was selected where no DRG existed to manage a specific toxicity. In the outpatient setting, costs were identified for medications, procedures, and physician consultations.

In Italy, inpatient costs were an average of the six most populous provinces in Italy using the regional tariffs per DRG. The provinces were Piemonte, Lombardia, Veneto, Lazio, Sicilia and Campania. The most recent tariffs were published in 2009 by the Agenzia Nazionale per i Servizi Sanitari Regionali (AgeNaS), and were assumed therefore to be in current use without further tariff inflation [18]. The outpatient costs were also average regional tariffs from AgeNaS [19]. The costs for platelets and blood used in transfusions came from the Ministero della Salute [20]. Ex-factory drug prices were obtained from the Agenzia Italiana del Farmaco [21].

In Spain, outpatient and inpatient costs were obtained from the OBLIKUE database, which collects all healthcare costs available in Spain, both from government databases and from the literature, and designates the best available cost information per indication [22]. As the government collects limited cost information, while government values were preferred, if unavailable, the best literature source was used. Drug prices were collected from the Ministry of Health’s INTEGRA database [23].

In Germany, the inpatient costs were derived from the Universitätsklinikum Münster, using the DRG Research Group Medizinisches Management search tool [24]. The outpatient costs came from The Kassenärztliche Bundesvereinigung Einheitlicher Bewertungsmaßstab (EBM), which is maintained by the Ambulatory Care Committee, a part of the Federal Joint Committee [25]. The prescription drug costs were derived from the Barmer GEK Arzneimittelreport and Lauertaxe database [26, 27]. The Arzneimittelreport was used to determine the most common drug for a specific indication and the Lauertaxe database was used to determine the price of the drug.

French inpatient and drug costs were found in public price lists maintained by the Agence Technique de l’information sur l’Hospitalisation (ATIH) [28]. Outpatient procedure costs were from the Sécurité Sociale l’Assurance Maladie en ligne Classification Commune des Actes Médicaux, and lab costs were from the Sécurité Sociale l’Assurance Maladie en ligne Table Nationale de codage de Biologie [29, 30].

In the Netherlands, the Tariefapplicatie from the Dutch Healthcare Authority (NZa) was used to identify inpatient and outpatient costs [31]. This source provides costs for different hospitals, so the lowest cost was selected for this exercise. Costs for consultations were from the Institute for Medical Technology Assessment at Erasmus University [32]. The cost for oxygen was from Praxisdienst [33]. Z-Index provided drug costs except in cases where products were not listed, in which case costs were obtained from Medicijnkosten [34, 35].

In the UK, inpatient, short stay, and outpatient costs came from the National Health Service (NHS) National Schedule of Referenced Costs, which reflect the average actual costs to the hospital to provide the activity, and is the preferred source for payer perspective costs in the UK [36]. The cost of granulocyte colony-stimulating factor (GCSF) was from the NHS Trust. Other drug costs were assumed to be included in the fee for an oncology consultation based on dispensing location and consultation fee definitions.

For Australia, inpatient costs were found in the Australian Government Department of Health and Ageing Medicare Benefits Schedule Book [37]. Outpatient costs were from the Australian Government Department of Health and Ageing National Public Cost Weight Tables [38]. Drug costs were from the Pharmaceutical Benefits Scheme (PBS) [39].

For Canada, inpatient costs were from the Ontario Case Costing Initiative (OCCI)—OCCI costing analysis tool [40]. For costs not available in OCCI due to lack of data, inpatient costs were from the Canadian Institute for Health Information Patient Cost Estimator [41]. Outpatient costs were from the Schedule of Benefits, Physician Services under the Health Insurance Act [42]. Drug costs were from the IMS Brogan database using the Association Québécoise des Pharmaciens Propriétaires (AQPP) prices [43].

To calculate outpatient and inpatient treatment of each AE, unit costs were multiplied by default resource utilization suggested by the clinical experts. A sensitivity analysis was conducted by applying the unit costs to non-base case resource utilization patterns in order to explore the range suggested by the interviews.

## Results

Forty-five publications on the pre-determined therapies were derived from the literature search. From these studies, a high-quality phase 3 study reporting grade 3 or 4 AEs for each line of therapy was chosen. But if the line of therapy was unclear or if a particular line of therapy had only phase 2 results published, phase 2 results were accepted; this was the case for first line ipilimumab and second line IL-2 and vemurafenib. Details of selected papers are reported in Table 1.

**Table 1:** Summary of clinical studies on the treatment of metastatic melanoma for which grade 3 or 4 AEs were reported

Comparator	Source study	Study setting	Phase	Sample size (*n*)	Treatment dose
Dabrafenib	Hauschild [46]	1st line	3	187	150 mg twice daily orally
Dacarbazine	Patel [47]	1st line	3	430	1000 mg/m^2^ on day 1 every 3 weeks
Fotemustine	Avril [48]	1st line	3	112	Weekly for 3 consecutive weeks at 100 mg/m^2^ followed by a 5-week rest period, with maintenance therapy for nonprogressive patients of 100 mg/m^2^ every 3 weeks
IL-2	Tarhini [49]	2nd line	2	26	600,000 IU/kg per dose for a maximum of 14 doses per cycle with a 1-week rest period between cycles. Stable or responding patients were offered an additional course after 6–8 weeks
Ipilimumab	Hersh [50]	1st line	2	37	3 mg/kg once every 4 weeks for four treatments
Ipilimumab	Hodi [8]	2nd line	3	137	3 mg/kg once every 3 weeks for four treatments
Temozolomide	Patel [47]	1st line	3	429	Orally once a day at a dose of 150 mg/m^2^/day for seven consecutive days every 2 weeks
Trametanib	Flaherty [51]	2nd line	3	214	2 mg twice daily orally
Vemurafenib	Chapman [52]	1st line	3	337	960 mg twice daily orally
Vemurafenib	Sosman [53]	2nd line	2	132	960 mg twice daily orally

A total of 30 (grade 3 or 4) AEs were identified from these publications, excluding toxicities that would not receive active management. Following the pilot interviews, resource utilization patterns indicated that some AEs would require a similar management approach or magnitude of treatment. As a result, 17 clusters were created from the list of AEs and the toxicity considered to be the most clinically significant was selected as a proxy for the others; total toxicity management costs were only estimated for the proxy toxicity. The full list of AEs by their groupings is presented in Table 2.

**Table 2:** List of toxicities grouped by general approach or magnitude of required management

Pruritus **Rash**	**Hypertension** Hypotension Peripheral edema Tachycardia
**Cutaneous squamous cell carcinoma** Keratocanthoma	Colitis (immune related) **Diarrhea (immune related)**
Nausea **Vomiting**	Constipation **Diarrhea**
**Hypophysitis**	**Dyspnea**
**Febrile neutropenia**	**Peripheral neuropathy**
Leukopenia Lymphopenia **Neutropenia** Thrombocytopenia	Asthenia Arthralgia **Fever**
**Elevated liver enzymes**	**Infection**
**Palmar-plantar hyperkeratosis**	**Headache**
**Anemia**	

Bold AEs represent the group proxy, followed by other AEs in the group

The most common grade 3 and 4 AEs included neutropenia, vomiting and anemia for chemotherapy agents. Vemurafenib was most commonly associated with cutaneous squamous cell carcinoma (CSCC)/keratocanthroma, rash and elevated liver enzymes and dabrafenib was more commonly associated with CSCC and pyrexia (fever). Hypertension and rash were most often related to trametinib. Common ipilimumab AEs were immune-related diarrhea/colitis, dyspnea, anemia, vomiting, and hypophysitis less frequently.

Base case outpatient costs are in Table 3. Inpatient treatment costs for the management of AEs are reported in Table 4. It was assumed that the inpatient cost for an AE was the same in the grade 3 and grade 4 settings.

**Table 3:** Outpatient total costs required to manage AEs due to treatment for metastatic melanoma

Adverse event	Grade	Italy	Spain	Germany	France	Netherlands	UK	Australia	Canada
Anemia	3	€1329	€1443	€46	€1285	€936	£730	$890	$370
	4	€1281^a^	€1443	€46	€1285^b^	€936	£730	$890	$370
Cutaneous squamous cell carcinoma	3	€297	€297	€406	€71	€1063	£720	$424	$205
Diarrhea	3	€46	€134	€46	€33	€86	£251	$91	$162
	4	€46	€134^b^	€46	€33	€86	£126^a^	$91	$162
Diarrhea (immune related)	3	€30	€134	€46	€29	€86	£251	$1121	$162
Dyspnea	3	€23	€99	€46	€156	€188	£251	$129^b^	$194
	4	€25	€99^b^	^c^	€156^b^	^d^	^d^	$393^b^	$194
Elevated liver enzymes	3	€47	€97	€46	€28	€79	£251	$304	$160
	4	€47	€97	€46	€28	€79	£251	$304	$160
Febrile neutropenia	3	€436	€598	€46	€29	€81	^d^	^d^	$258
	4	€436	€598	€46	€29	€81	^d^	^d^	$258
Fever	3	€21	€104	€46	€28	€82	£251	$94	$161
Headache	3	€255	€98	€46	€314	€82	£251	$99	$227
Hypertension	3	€46	€104	€61	€30	€79	£251	$97	$164
Hypophysitis	3	€326	€460	€46	€107	€465	£251	$283	$168
Infection	3	€34	€99	^c^	€67	€81	£251	^d^	$161
	4	€34	€99^b^	^c^	€67	^d^	£251	^d^	$161
Neutropenia	3	€89	€598	€46	€28	€79	£251	$141	$160
	4	€497	€755	€46	€28	€79	^d^	$210	$160
Palmar plantar hyperkeratosis	3	€43	€173	€46	€28	€158	£126	$203	$160
Peripheral neuropathy	3	€173	€1289	€46	€28	€83	£126	$152	$183
	4	€173	€1289	€46	€28	€83	£251	$218	$210
Rash	3	€47	€184	€46	€32	€86	£251	$380	$171
	4	€47	€184^b^	^c^	€32	€82^e^	£251	$373^a^	$162^f^
Vomiting	3	€64	€132	€76	€31	€80	£251	$147	$239
	4	€64	€132	^c^	€31	€80	£251	^d^	$239

^a^Grade 4 outpatient treatment is less expensive versus grade 3 because more physicians recommended patients be hospitalized in grade 4; ^b^ Although clinicians provided outpatient resource use, they indicated that patients would always be hospitalized; ^c^ Never seen; ^d^ 100 % inpatient admission; ^e ^ Physicians suggested more prescription products for grade 3 versus grade 4; ^f^ Topical steroids for grade 3 were more expensive than oral steroids for grade 4, as cream can only be purchased per tube

**Table 4:** Inpatient costs of treating grade 3 or 4 events due to treatment for metastatic melanoma

Adverse event	Italy	Spain	Germany	France	Netherlands	UK	Australia	Canada
Anemia	€2667	€1801	€2367	€2000	€2839	£2246	$4380	$5181
Cutaneous squamous cell carcinoma	€1589	€1221	€1544	€1416	€2122	£1692	$2379	$8934
Diarrhea	€1456	€4113	€1348	€1585	€1456	£4284	$4572	$4320
Diarrhea (immune related)	€1456	€4113	€1348	€1585	€1456	£4284	$4572	$4320
Dyspnea	€1689	€1755	€9077	€1466	€1431	£1209	$3671	$5506
Elevated liver enzymes	€2159	€3356	€1809	€6913	€1305	£1819	$6594	$8030
Febrile neutropenia	€2357	€5480	€2388	€2000	€2152	£4444	$5224	$7843
Fever	€3433	€2822	€1686	€1658	€1411	£1598	$4375	$5008
Headache	€2366	€2489	€1644	€1002	€1718	£1372	$1935	$3479
Hypertension	€1573	€2405	€2246	€1619	€1702	£3852	$4711	$7028
Hypophysitis	€1589	€10,265	€1979	€5316	€1683	£2417	$7231	$9735
Infection	€3433	€4477	€2099	€3018	€1806	£1918	$7199	$6563
Neutropenia	€2357	€1529	€2388	€2000	€877	£2194	$5224	$7843
Palmar plantar hyperkeratosis	€1308	€5121	€1544	–	€1606	£1692	$2654	$4177
Peripheral neuropathy	€1972	€4144	€2004	€2625	€6977	£2617	$4923	$9472
Rash	€1308	€2087	€1544	–	€1764	£1692	$2654	$3223
Vomiting	€1456	€1755	€1348	€1585	€2045	£1702	$4572	$3543

Results vary by country, reflecting variation in unit costing across country borders, but trends appeared indicating that hospitalization, outpatient procedures, and certain high-cost medications lead to expensive AE management. Anemia required high-cost outpatient care across all countries except for Germany due to use of erythropoietin and/or blood transfusions. These costs range from €936 (Netherlands) to €1443 (Spain), £730 (UK), CAN$370 (Canada), and AUS$890 (Australia). Aside from anemia, other costly outpatient AEs included CSCC in the Netherlands (€1063), Germany (€406) and UK (£720), febrile/afebrile neutropenia for Italy and Spain (€497–755), peripheral neuropathy for Spain (€1289) and immune related diarrhea for Australia (AUS$1121). In the Netherlands and Germany, the necessary outpatient excision of CSCC drove the higher costs for that treatment, while febrile/afebrile neutropenia expenditure was related to use of granulocyte colony-stimulating factors (GCSF). Specialist consultations resulted in the high cost for peripheral neuropathy in Spain while the cost for immunosuppressant treatment contributed to the high cost of immune-related diarrhea in Australia. See Table 3 for detailed cost results.

Hospitalizations were more costly in general, but the most expensive AEs to treat in the inpatient setting include hypophysitis in Spain (€10,265), Canada (CAN$9735), and Australia (AUS$7231), dyspnea in Germany (€9077), febrile neutropenia in the UK (£4444), elevated liver enzymes in France (€6913), fever in Italy (€3433), and peripheral neuropathy in Netherlands (€6977). Febrile neutropenia was in the top three most expensive hospitalizations in multiple countries, as well, including Germany, Spain, Netherlands, and UK (ranging from €2152 to €5480, or £4444 in UK). All Euro-zone hospitalizations exceeded €1000 per episode, while the lowest costs were approximately £1300 in UK, AUS$1900 in Australia, and CAN$3200 in Canada. Detailed results can be found in Table 4.

As with base case results, country variation in sensitivity analysis results exists as well. Due to cost-conserving approaches to treatment, low ranges did not differ importantly from base case results across the countries, but more expensive options were suggested for management of certain AEs, with some notable trends, such as higher cost medications suggested in lieu of base case treatment. Cost of treating febrile or afebrile neutropenia could rise based on the use of GCSF during treatment in Italy, Germany, France, and the UK, with associated excess costs of ~€900, ~€1400, ~€3800, and ~£240, respectively. In Canada, use of antibiotics against febrile neutropenia could inflate costs by approximately CAN$250. Use of erythropoietin products to treat anemia also resulted in higher costs in Italy, France, Spain, and the Netherlands (over €1000 more). The use of mycophenolate in Australia could increase the cost of treating rash and palmar plantar hyperkeratosis by as much as AUS$1000, as well.

Adding specialist consultations, facility stays, or procedures raised the total costs per AE as well. Additional consultations increased the cost to treat neutropenia and immune-related diarrhea in Canada by over CAN$1000 per episode. Short facility stays in the UK led to higher costs of managing grade 4 diarrhea and neutropenia of approximately £550-750. Transfusion procedures (thrombocyte or leukocyte) were costly additions to treatment for hematological toxicities in the Netherlands and France (€857 and €629, respectively).

## Discussion

Managing AEs resulting from metastatic melanoma treatment can incur significant costs, even accounting for variation in country-level or individual differences in treatment approach. Payer-perspective costs of total resources administered or recommended by a medical oncologist or dermatologist (i.e., not including resources required for continuing care by another specialist), ranged from the cost of an excess physician visit to high hospitalization costs. This is a conservative estimate of the resources used to treat toxicities related to metastatic melanoma treatments; if other specialists are consulted to manage specific events, for instance, additional resources may be required that this study does not capture.

Although differences existed across countries in itemized costs and treatment approaches, as well as in extent of additional specialist consultation (see Appendix for detailed resource utilization and cost values), some AE management cost trends were apparent. In the outpatient setting, anemia was one of the most costly AEs to treat in most countries examined in this study. CSCC and immune-related diarrhea were also costly in several countries. In the inpatient setting, hypophysitis, elevated liver enzymes, peripheral neuropathy, dyspnea, diarrhea, CSCC, and febrile neutropenia incurred higher costs relative to other AEs related to melanoma treatments. AEs such as headache and rash were among the least costly hospitalizations. These trends reflect the higher unit costs associated with hospitalization, certain expensive medications, and procedures. Some of the common AEs associated with ipilimumab (hypophysitis, dyspnea, and diarrhea) and vemurafenib/dabrafenib (CSCC and elevated liver enzymes) are among the most expensive AEs.

A recent Italian retrospective cohort study investigated the cost of unresectable stage III or stage IV melanoma, providing some support for our findings in the Italian setting [44]. Although Maio et al. do not report costs per specific AE, they do investigate claims for drug therapies associated with treating large categories of AEs that occur in at least 5 % of the population. Categories of AEs in the Maio analysis are based on type of drug therapy required for treatment, including such drugs as ondansetron, dexamethasone, filgrastim, lenograstim and pegfilgrastim. Maio et al. show similar prices per day for each drug as in the current analysis, applied for slightly longer treatment durations than in our analysis based on the average duration of therapy in their database. Although our analysis supplements drug costs with other outpatient and inpatient costs explicitly related to the adverse event, both analyses demonstrate that managing AEs can require substantial resources in Italy, suggesting that a melanoma regimen with fewer and less costly AEs will ease some of the burden faced by the patients with regionally or distantly metastatic melanoma and the clinical community.

This study was designed to demonstrate that managing each AE associated with melanoma treatment can be costly; specific values presented in this study for multiple country settings can be used in future analyses, including modeling to assess the overall burden of managing AEs over the course of therapy. Although not covered in this study, a recent US retrospective database analysis investigated the overall AE costs associated with several different melanoma therapies (vemurafenib, ipilimumab, dacarbazine, paclitaxel and temozolomide). The authors found that hemic and lymphatic disorders and effects incurred the highest costs across therapies. Adjusted mean costs for AEs were highest for dacarbazine and lowest for vemurafenib among the treatments considered [45].

This study does have some limitations, however. The study does not include pembrolizumab or nivolumab as these products were not approved at the time of study completion; however, AE profiles for these drugs do not require significant additions to the already comprehensive list assessed in this analysis. Moreover, although clinical trials may not capture the set of real-world AEs, this source was considered the most comprehensive and consistent source across therapies under consideration and thus used to determine the list considered in this study. Also, the resource use estimates in this analysis may not represent the full spectrum of clinical practice patterns or duration of required management across all patients. This study depended on physician interviews to derive resource use information, and five clinical experts may not represent a nation’s practice patterns as a whole. However, their responses serve to indicate some approaches to management, and the use of clinician agreement for a base case estimate with sensitivity ranges to explore variation creates a picture of the magnitude of financial impact in each country. Additionally, only resources that would be used from the medical oncologist or dermatologist’s perspective were included. These estimates may not include additional resources a specialist requires to continue management of an AE, leading to potential underestimation of the total costs. However, higher costs would only further support the suggestion of sizeable AE-related cost burden. Regarding the use of specific data sources, the cost estimates used in Spain included both government costs and literature-based values, as derived from the OBLIKUE database. Given the limited nature of cost information in Spain, the OBLIKUE database compiles the best available data sources, and therefore the costs used in this study constitute the best representation of the public payer perspective in Spain. Finally, this study does not explore any indirect costs associated with advanced or metastatic melanoma, as this analysis took the payer perspective in each country. Therefore, costs identified here likely underestimate the true burden of melanoma in these countries.

In light of these limitations, the findings from this analysis should be interpreted with caution; results can be considered an indication of the extent of the economic issue, as well as an initial understanding of the AE-specific costs per AE that payers may face. As one of the only close examinations into the size of global impact per AE, this initial financial picture can help inform future evaluations of the clinical and cost-effectiveness of treating patients with metastatic melanoma while serving as an impetus for further study. As information continues to accrue for the newest therapies in the coming months and years, opportunities will exist to complement this effort via future analysis of real-world resource use and costs related to treating toxicities as a result of metastatic melanoma therapy.

## Electronic supplementary material

Below is the link to the electronic supplementary material.

Supplementary material 1 (DOCX 107 kb)
