# Supplementary material for: Economic burden of toxicities associated with treating metastatic melanoma in eight countries
Source: Eur J Health Econ. 2015 Dec 31;18(1):49–58. doi: 10.1007/s10198-015-0757-y (PMC5209401; doi:10.1007/s10198-015-0757-y)
Supplement: Supplementary file 1 — Supplementary material 1 (DOCX 107 kb) [file 10198_2015_757_MOESM1_ESM.docx]

Title: Economic burden of toxicities associated with treating metastatic melanoma in eight countries

Journal name: European Journal of Health Economics

Authors: Elizabeth Wehler, Zhongyun Zhao, S. Pinar Bilir, Julie Munakata, Beth Barber

Corresponding author:

Elizabeth Wehler

[bwehler@us.imshealth.com](mailto:bwehler@us.imshealth.com)

Table 1: Resource Use and Costs for Italy

| **Toxicity** | **Grade** | **Resource** | **Total resource cost** | **Total resource cost per toxicity** | **Comments** |
| --- | --- | --- | --- | --- | --- |
| Anemia | 3 | Oncology consultation | € 21.39 | € 1,329.01 |  |
|  |  | Blood test | € 67.19 |  |  |
|  |  | Blood transfusion | € 202.33 |  |  |
|  |  | Darbepoetin alfa (Aranesp) | € 1,038.09 |  | 500 mcg every 3 weeks |
|  | 4 | Oncology consultation | € 21.39 | € 1,281.01 |  |
|  |  | Blood transfusion | € 202.33 |  |  |
|  |  | Darbepoetin alfa (Aranesp) | € 1,038.09 |  | 500 mcg every 3 weeks |
|  |  | Iron and vitamins | € 19.19 |  |  |
|  |  |  |  |  |  |
| Cutaneous squamous cell carcinoma | 3 | Oncology consultation | € 21.39 | € 296.52 |  |
|  |  | Dermatology consultation | € 21.39 |  |  |
|  |  | Excision | € 253.73 |  |  |
|  |  |  |  |  |  |
| Diarrhea | 3 | Oncology consultation | € 21.39 | € 46.16 |  |
|  |  | Gastro consultation | € 21.39 |  |  |
|  |  | Hydration | € 1.83 |  |  |
|  |  | Loperamide | € 1.31 |  | 3 to 4 2 mg tablets daily for 3 to 4 days |
|  |  | Metoclopramide | € 0.23 |  | 1 10 mg vial, once |
|  |  |  |  |  |  |
|  | 4 | Oncology consultation | € 21.39 | € 46.16 |  |
|  |  | Gastro consultation | € 21.39 |  |  |
|  |  | Hydration | € 1.83 |  |  |
|  |  | Loperamide | € 1.31 |  | 3 to 4 2 mg tablets daily for 3 to 4 days |
|  |  | Metoclopramide | € 0.23 |  | 1 10 mg vial, once |
|  |  |  |  |  |  |
| Diarrhea (immune-related) | 3 | Oncology consultation | € 21.39 | € 29.50 |  |
|  |  | Loperamide | € 1.31 |  | 3 to 4 2 mg tablets daily for 3 to 4 days |
|  |  | Methylprednisone (Solumedrol) | € 2.44 |  | 6 mg tablets, twice daily for 4 days |
|  |  | Prednisone | € 2.53 |  | 25 mg, once daily for 7 days |
|  |  | Hydration | € 1.83 |  |  |
|  |  |  |  |  |  |
| Dyspnea | 3 | Oncology consultation | € 21.39 | € 22.72 |  |
|  |  | Betametasone | € 1.33 |  | 2.5 mg, once daily for 4-5 days |
|  |  |  |  |  |  |
|  | 4 | Oncology consultation | € 21.39 | € 24.81 |  |
|  |  | Betametasone | € 1.33 |  | 2.5 mg, once daily for 4-5 days |
|  |  | Aminomal (Aminophylline) | € 2.09 |  | 350 mg, once daily for 4-5 days |
|  |  |  |  |  |  |
| Elevated liver enzymes | 3 | Oncology consultation | € 21.39 | € 47.20 |  |
|  |  | Liver function test | € 19.19 |  |  |
|  |  | Dexamethasone | € 4.59 |  | 6 mg, once daily for 4-5 days |
|  |  | Tranexamic acid (Tranex) | € 2.03 |  | 1 500 mg vial, 3 times for 1 day |
|  |  |  |  |  |  |
|  | 4 | Oncology consultation | € 21.39 | € 47.20 |  |
|  |  | Liver function test | € 19.19 |  |  |
|  |  | Dexamethasone | € 4.59 |  | 6 mg, once daily for 4-5 days |
|  |  | Tranexamic acid (Tranex) | € 2.03 |  | 1 500 mg vial, 3 times for 1 day |
|  |  |  |  |  |  |
| Febrile neutropenia | 3 | Oncology consultation | € 21.39 | € 436.43 |  |
|  |  | Lemograstim | € 402.85 |  | 33.6 MUI vial daily for 5 days |
|  |  | Ciproxin | € 12.18 |  | 500 mg twice daily for 7 days |
|  |  |  |  |  |  |
|  | 4 | Oncology consultation | € 21.39 | € 436.43 |  |
|  |  | Lemograstim | € 402.85 |  | 33.6 MUI vial daily for 5 days |
|  |  | Ciproxin | € 12.18 |  | 500 mg twice daily for 7 days |
|  |  |  |  |  |  |
|  |  |  |  |  |  |
| Fever | 3 | Oncology consultation | € 21.39 | € 21.08 |  |
|  |  |  |  |  |  |
| Headache | 3 | Oncology consultation | € 21.39 | € 255.02 |  |
|  |  | Prednisone | € 7.22 |  | 25 mg, twice daily for 10 days |
|  |  | Brain MRI | € 226.41 |  |  |
|  |  |  |  |  |  |
| Hypertension | 3 | Oncology consultation | € 21.39 | € 46.43 |  |
|  |  | Cardiology consultation | € 21.39 |  |  |
|  |  | Amlodipine (Norvasc) | € 3.64 |  | 5 mg daily for 30 days |
|  |  |  |  |  |  |
| Hypophysitis | 3 | Oncology consultation | € 21.39 | € 325.82 |  |
|  |  | Blood test | € 67.19 |  |  |
|  |  | Prednisone | € 10.83 |  | 25 mg daily for 30 days |
|  |  | MRI of the pineal gland | € 226.41 |  |  |
|  |  |  |  |  |  |
| Infection | 3 | Oncology consultation | € 21.39 | € 33.58 |  |
|  |  | Ciproxin | € 12.18 |  | 500 mg twice daily for 7 days |
|  |  |  |  |  |  |
|  | 4 | Oncology consultation | € 21.39 | € 33.58 |  |
|  |  | Ciproxin | € 12.18 |  | 500 mg twice daily for 7 days |
|  |  |  |  |  |  |
| Neutropenia | 3 | Oncology consultation | € 21.39 | € 88.58 |  |
|  |  | Blood test | € 67.19 |  |  |
|  |  |  |  |  |  |
|  | 4 | Oncology consultation | € 21.39 | € 497.27 |  |
|  |  | Platelet transfusion | € 471.28 |  |  |
|  |  | Dexamethasone (Soldesam) | € 4.59 |  | 6 mg daily for 4-5 days |
|  |  |  |  |  |  |
| Palmar plantar hyperkeratosis | 3 | Oncology consultation | € 21.39 | € 42.79 |  |
|  |  | Dermatologist consultation | € 21.39 |  |  |
|  |  |  |  |  |  |
| Peripheral neuropathy | 3 | Oncology consultation | € 21.39 | € 172.95 |  |
|  |  | Neurology consultation | € 21.39 |  |  |
|  |  | Electromyography | € 11.11 |  |  |
|  |  | Pregabalin (Lyrica) | € 119.04 |  | 75 mg twice daily for 7 days, then 150 mg twice daily for 45 days |
|  |  |  |  |  |  |
|  | 4 | Oncology consultation | € 21.39 | € 172.95 |  |
|  |  | Neurology consultation | € 21.39 |  |  |
|  |  | Electromyography | € 11.11 |  |  |
|  |  | Pregabalin (Lyrica) | € 119.04 |  | 75 mg twice daily for 7 days, then 150 mg twice daily for 45 days |
|  |  |  |  |  |  |
| Rash | 3 | Oncology consultation | € 21.39 | € 46.64 |  |
|  |  | Cetirizine (Zyrtec) | € 1.32 |  | 10 mg daily for 5 days |
|  |  | Prednisone | € 2.53 |  | 0.5 mg/kg daily for 5 days |
|  |  | Dermatology consultation | € 21.39 |  |  |
|  |  |  |  |  |  |
|  | 4 | Oncology consultation | € 21.39 | € 46.64 |  |
|  |  | Cetirizine (Zyrtec) | € 1.32 |  | 10 mg daily for 5 days |
|  |  | Prednisone | € 2.53 |  | 0.5 mg/kg daily for 5 days |
|  |  | Dermatology consultation | € 21.39 |  |  |
|  |  |  |  |  |  |
| Vomiting | 3 | Oncology consultation | € 21.39 | € 64.16 |  |
|  |  | Ondansetron (Zofran) | € 40.94 |  | 8 mg three times daily for 3 days |
|  |  | Hydration | € 1.83 |  |  |
|  |  |  |  |  |  |
|  | 4 | Oncology consultation | € 21.39 | € 64.16 |  |
|  |  | Ondansetron (Zofran) | € 40.94 |  | 8 mg three times daily for 3 days |
|  |  | Hydration | € 1.83 |  |  |

Table 2: Resource Use and Costs for Spain

| **Toxicity** | **Grade** | **Resource** | **Total resource cost** | **Total resource cost per toxicity** | **Comments** |
| --- | --- | --- | --- | --- | --- |
| Anemia | 3 | Oncology consultation | € 97.07 | € 1,443.21 |  |
|  |  | Epoetin alfa (Eprex) | € 1,346.14 |  | 40,000 IU each week for 4 weeks |
|  |  |  |  |  |  |
|  | 4 | Oncology consultation | € 97.07 | € 1,443.21 |  |
|  |  | Epoetin alfa (Eprex) | € 1,346.14 |  | 40,000 IU each week for 4 weeks |
|  |  |  |  |  |  |
| Cutaneous squamous cell carcinoma | 3 | Oncology consultation | € 97.07 | € 296.59 |  |
|  |  | Dermatology consultation | € 75.73 |  |  |
|  |  | Outpatient excision | € 123.78 |  |  |
|  |  |  |  |  |  |
| Diarrhea | 3 | Oncology consultation | € 97.07 | € 134.25 |  |
|  |  | Methylprednisone | € 12.64 |  | 70 mg/day for 10 days |
|  |  | Physiological serum | € 24.54 |  | 2,000 ml/day for 5-10 days |
|  |  |  |  |  |  |
|  | 4 | Oncology consultation | € 97.07 | € 134.25 |  |
|  |  | Methylprednisone | € 12.64 |  | 70 mg/day for 10 days |
|  |  | Physiological serum | € 24.54 |  | 2,000 ml/day for 5-10 days |
|  |  |  |  |  |  |
| Diarrhea (immune-related) | 3 | Oncology consultation | € 97.07 | € 134.25 |  |
|  |  | Methylprednisone | € 12.64 |  | 70 mg/day for 10 days |
|  |  | Physiological serum | € 24.54 |  | 2,000 ml/day for 5-10 days |
|  |  |  |  |  |  |
| Dyspnea | 3 | Oncology consultation | € 97.07 | € 98.90 |  |
|  |  | Amoxicillin | € 1.83 |  | 500 mg three times a day for 7 days |
|  |  |  |  |  |  |
|  | 4 | Oncology consultation | € 97.07 | € 98.90 |  |
|  |  | Amoxicillin | € 1.83 |  | 500 mg three times a day for 7 days |
|  |  |  |  |  |  |
| Elevated liver enzymes | 3 | Oncology consultation | € 97.07 | € 97.07 |  |
|  |  |  |  |  |  |
|  | 4 | Oncology consultation | € 97.07 | € 97.07 |  |
|  |  |  |  |  |  |
| Febrile neutropenia | 3 | Oncology consultation | € 97.07 | € 598.34 |  |
|  |  | Filgrastim (Neupogen) | € 499.44 |  | 5 mcg/kg/day for 1 week |
|  |  | Amoxicillin | € 1.83 |  | 500 mg three times a day for 7 days |
|  |  |  |  |  |  |
|  | 4 | Oncology consultation | € 97.07 | € 598.34 |  |
|  |  | Filgrastim (Neupogen) | € 499.44 |  | 5 mcg/kg/day for 1 week |
|  |  | Amoxicillin | € 1.83 |  | 500 mg three times a day for 7 days |
|  |  |  |  |  |  |
| Fever | 3 | Oncology consultation | € 97.07 | € 104.45 |  |
|  |  | Paracetamol | € 7.38 |  | 1 g three times a day for 3-4 days |
|  |  |  |  |  |  |
| Headache | 3 | Oncology consultation | € 97.07 | € 97.78 |  |
|  |  | Paracetamol | € 0.70 |  | 1 g three times a day for 3-4 days |
|  |  |  |  |  |  |
| Hypertension | 3 | Oncology consultation | € 97.07 | € 103.79 |  |
|  |  | Captopril | € 4.55 |  | 100 mg/day for 30 days |
|  |  | Losartan | € 2.16 |  | 50 mg/day for 30 days |
|  |  |  |  |  |  |
| Hypophysitis | 3 | Oncology consultation | € 97.07 | € 460.19 |  |
|  |  | 2 Endocrinology consultations | € 299.10 |  |  |
|  |  | Thyroid hormone replacement | € 10.62 |  |  |
|  |  | Dexamethasone | € 53.40 |  | 8 mg/day for 10-15 days |
|  |  |  |  |  |  |
| Infection | 3 | Oncology consultation | € 97.07 | € 98.56 |  |
|  |  | Amoxicillin | € 2.22 |  | 500 mg three times daily for 7-10 days |
|  |  |  |  |  |  |
|  | 4 | Oncology consultation | € 97.07 | € 98.56 |  |
|  |  | Amoxicillin | € 2.22 |  | 500 mg three times daily for 7-10 days |
|  |  |  |  |  |  |
| Neutropenia | 3 | Oncology consultation | € 97.07 | € 598.34 |  |
|  |  | Filgrastim (Neupogen) | € 499.44 |  | 5 mcg/kg/day for one week |
|  |  | Amoxicillin | € 1.83 |  | 500 mg three times daily for 7 days |
|  |  |  |  |  |  |
|  | 4 | Oncology consultation | € 97.07 | € 755.05 |  |
|  |  | Filgrastim (Neupogen) | € 499.44 |  | 5 mcg/kg/day for one week |
|  |  | Amoxicillin | € 1.83 |  | 500 mg three times daily for 7 days |
|  |  | Hematology consultation | € 156.71 |  |  |
|  |  |  |  |  |  |
| Palmar plantar hyperkeratosis | 3 | Oncology consultation | € 97.07 | € 172.80 |  |
|  |  | Dermatologist consultation | € 75.73 |  |  |
|  |  |  |  |  |  |
| Peripheral neuropathy | 3 | Oncology consultation | € 97.07 | € 1,289.31 |  |
|  |  | 5 Neurology consultations | € 1,192.23 |  |  |
|  |  |  |  |  |  |
|  | 4 | Oncology consultation | € 97.07 | € 1,289.31 |  |
|  |  | 5 Neurology consultations | € 1,192.23 |  |  |
|  |  |  |  |  |  |
| Rash | 3 | Oncology consultation | € 97.07 | € 184.02 |  |
|  |  | Celestamine | € 11.13 |  | 2 mg 3-6 times daily for one month |
|  |  | Dermatology consultation | € 75.17 |  |  |
|  |  |  |  |  |  |
|  | 4 | Oncology consultation | € 97.07 | € 184.02 |  |
|  |  | Celestamine | € 11.22 |  | 2 mg 3-6 times daily for one month |
|  |  | Dermatology consultation | € 75.17 |  |  |
|  |  |  |  |  |  |
| Vomiting | 3 | Oncology consultation | € 97.07 | € 131.52 |  |
|  |  | Ondansetron (Zofran) | € 28.68 |  | 8 mg, twice daily for 5 days |
|  |  | Dexamethasone | € 5.76 |  | 4 mg, twice daily for 2-3 days |
|  |  |  |  |  |  |
|  | 4 | Oncology consultation | € 97.07 | € 131.52 |  |
|  |  | Ondansetron (Zofran) | € 28.68 |  | 8 mg, twice daily for 5 days |
|  |  | Dexamethasone | € 5.76 |  | 4 mg, twice daily for 2-3 days |

Table 3: Resource Use and Costs for Germany

| **Toxicity** | **Grade** | **Resource** | **Total resource cost** | **Total resource cost per toxicity** | **Comments** |
| --- | --- | --- | --- | --- | --- |
| Anemia | 3 | Oncology consultation | € 45.98 | € 45.98 |  |
|  |  |  |  |  |  |
|  | 4 | Oncology consultation | € 45.98 | € 45.98 |  |
|  |  |  |  |  |  |
| Cutaneous squamous cell carcinoma | 3 | Oncology consultation | € 45.98 | € 406.09 |  |
|  |  | Excision | € 360.11 |  |  |
|  |  |  |  |  |  |
| Diarrhea | 3 | Oncology consultation | € 45.98 | € 45.98 |  |
|  |  |  |  |  |  |
|  | 4 | Oncology consultation | € 45.98 | € 45.85 |  |
|  |  |  |  |  |  |
| Diarrhea (immune-related) | 3 | Oncology consultation | € 45.98 | € 45.98 |  |
|  |  |  |  |  |  |
| Dyspnea | 3 | Oncology consultation | € 45.98 | € 45.98 |  |
|  |  |  |  |  |  |
|  |  |  |  |  |  |
|  | 4 | None |  | € 0.00 |  |
|  |  |  |  |  |  |
| Elevated liver enzymes | 3 | Oncology consultation | € 45.98 | € 45.98 |  |
|  |  |  |  |  |  |
|  | 4 | Oncology consultation | € 45.98 | € 45.98 |  |
|  |  |  |  |  |  |
| Febrile neutropenia | 3 | Oncology consultation | € 45.98 | € 45.98 |  |
|  |  |  |  |  |  |
|  | 4 | Oncology consultation | € 45.98 | € 45.98 |  |
|  |  |  |  |  |  |
| Fever | 3 | Oncology consultation | € 45.98 | € 45.98 |  |
|  |  |  |  |  |  |
| Headache | 3 | Oncology consultation | € 45.98 | € 45.98 |  |
|  |  |  |  |  |  |
| Hypertension | 3 | Oncology consultation | € 45.98 | € 60.89 |  |
|  |  | Metoprolol tartrate (Metobeta) | € 14.91 |  | 200 mg once daily for 30 days |
|  |  |  |  |  |  |
| Hypophysitis | 3 | Oncology consultation | € 45.98 | € 45.98 |  |
|  |  |  |  |  |  |
| Infection | 3 | None |  | € 0.00 |  |
|  |  |  |  |  |  |
|  | 4 | None |  | € 0.00 |  |
|  |  |  |  |  |  |
| Neutropenia | 3 | Oncology consultation | € 45.98 | € 45.98 |  |
|  |  |  |  |  |  |
|  | 4 | Oncology consultation | € 45.98 | € 45.98 |  |
|  |  |  |  |  |  |
| Palmar plantar hyperkeratosis | 3 | Oncology consultation | € 45.98 | € 45.98 |  |
|  |  |  |  |  |  |
| Peripheral neuropathy | 3 | Oncology consultation | € 45.98 | € 45.98 |  |
|  |  |  |  |  |  |
|  | 4 | Oncology consultation | € 45.98 | € 45.98 |  |
|  |  |  |  |  |  |
| Rash | 3 | Oncology consultation | € 45.98 | € 45.98 |  |
|  |  |  |  |  |  |
|  | 4 | None |  | € 0.00 |  |
|  |  |  |  |  |  |
| Vomiting | 3 | Oncology consultation | € 45.98 | € 75.50 |  |
|  |  | Granisetron (Kevatril) | € 29.52 |  | 1 mg once |
|  |  |  |  |  |  |
|  | 4 | None |  | € 0.00 |  |

Table 4: Resource Use and Costs for France

| **Toxicity** | **Grade** | **Resource** | **Total resource cost** | **Total resource cost per toxicity** | **Comments** |
| --- | --- | --- | --- | --- | --- |
| Anemia | 3 | Oncology consultation | € 28.19 | € 1,284.86 |  |
|  |  | Blood transfusion | € 628.72 |  |  |
|  |  | Epoetin alfa (Eprex) | € 627.95 |  | 150 IU/kg 3 times a week for 4 weeks |
|  |  |  |  |  |  |
|  | 4 | Oncology consultation | € 28.19 | € 1,284.86 |  |
|  |  | Blood transfusion | € 628.72 |  |  |
|  |  | Epoetin alfa (Eprex) | € 627.95 |  | 150 IU/kg 3 times a week for 4 weeks |
|  |  |  |  |  |  |
| Cutaneous squamous cell carcinoma | 3 | Oncology consultation | € 28.19 | € 71.40 |  |
|  |  | Excision | € 43.21 |  |  |
|  |  |  |  |  |  |
| Diarrhea | 3 | Oncology consultation | € 28.19 | € 32.71 |  |
|  |  | Imodium | € 4.52 |  | 4 mg twice daily for 2 days |
|  |  |  |  |  |  |
|  | 4 | Oncology consultation | € 28.19 | € 32.71 |  |
|  |  | Imodium | € 4.52 |  | 4 mg twice daily for 2 days |
|  |  |  |  |  |  |
| Diarrhea (immune-related) | 3 | Oncology consultation | € 28.19 | € 28.75 |  |
|  |  | Prednisone | € 0.56 |  | 40 mg once daily for 7 days |
|  |  |  |  |  |  |
| Dyspnea | 3 | Oncology consultation | € 28.19 | € 156.31 |  |
|  |  | CT scan | € 128.12 |  |  |
|  |  |  |  |  |  |
|  | 4 | Oncology consultation | € 28.19 | € 156.31 |  |
|  |  | CT scan | € 128.12 |  |  |
|  |  |  |  |  |  |
| Elevated liver enzymes | 3 | Oncology consultation | € 28.19 | € 28.19 |  |
|  |  |  |  |  |  |
|  | 4 | Oncology consultation | € 28.19 | € 28.19 |  |
|  |  |  |  |  |  |
| Febrile neutropenia | 3 | Oncology consultation | € 28.19 | € 28.92 |  |
|  |  | Amoxicillin | € 0.74 |  | 2 gr daily for 7 days |
|  |  |  |  |  |  |
|  | 4 | Oncology consultation | € 28.19 | € 28.92 |  |
|  |  | Amoxicillin | € 0.74 |  | 2 gr daily for 7 days |
|  |  |  |  |  |  |
| Fever | 3 | Oncology consultation | € 28.19 | € 28.19 |  |
|  |  |  |  |  |  |
| Headache | 3 | Oncology consultation | € 28.19 | € 313.56 |  |
|  |  | Brain MRI | € 285.38 |  |  |
|  |  |  |  |  |  |
| Hypertension | 3 | Oncology consultation | € 28.19 | € 29.86 |  |
|  |  | Furosemide | € 1.67 |  | 40 mg twice daily for 30 days |
|  |  |  |  |  |  |
| Hypophysitis | 3 | Oncology consultation | € 28.19 | € 107.40 |  |
|  |  | Prednisone | € 4.19 |  | 1 mg/kg daily for 30 days |
|  |  | Thyroid hormone replacement | € 0.83 |  | 1.7 mcg/kg/day for 30 days |
|  |  | Hormone tests | € 74.20 |  |  |
|  |  |  |  |  |  |
| Infection | 3 | Oncology consultation | € 28.19 | € 66.72 |  |
|  |  | Paracetamol | € 0.29 |  | 1 gr three times daily for 5 days |
|  |  | Ceftriaxone | € 38.25 |  | 1 gr daily for 5 days |
|  |  |  |  |  |  |
|  | 4 | Oncology consultation | € 28.19 | € 66.72 |  |
|  |  | Paracetamol | € 0.29 |  | 1 gr three times daily for 5 days |
|  |  | Ceftriaxone | € 38.25 |  | 1 gr daily for 5 days |
|  |  |  |  |  |  |
| Neutropenia | 3 | Oncology consultation | € 28.19 | € 28.19 |  |
|  |  |  |  |  |  |
|  | 4 | Oncology consultation | € 28.19 | € 28.19 |  |
|  |  |  |  |  |  |
| Palmar plantar hyperkeratosis | 3 | Oncology consultation | € 28.19 | € 28.19 |  |
|  |  |  |  |  |  |
| Peripheral neuropathy | 3 | Oncology consultation | € 28.19 | € 28.19 |  |
|  |  |  |  |  |  |
|  | 4 | Oncology consultation | € 28.19 | € 28.19 |  |
|  |  |  |  |  |  |
| Rash | 3 | Oncology consultation | € 28.19 | € 32.01 |  |
|  |  | Diprozone | € 3.83 |  | One tube |
|  |  |  |  |  |  |
|  | 4 | Oncology consultation | € 28.19 | € 32.01 |  |
|  |  | Diprozone | € 3.83 |  | One tube |
|  |  |  |  |  |  |
| Vomiting | 3 | Oncology consultation | € 28.19 | € 30.73 |  |
|  |  | Ondansetron (Zofran) | € 2.55 |  | 8 mg twice daily for 2 days |
|  |  |  |  |  |  |
|  |  |  |  |  |  |
|  | 4 | Oncology consultation | € 28.19 | € 30.73 |  |
|  |  | Ondansetron (Zofran) | € 2.55 |  | 8 mg twice daily for 2 days |

Table 5: Resource Use and Costs for the Netherlands

| **Toxicity** | **Grade** | **Resource** | **Total resource cost** | **Total resource cost per toxicity** | **Comments** |
| --- | --- | --- | --- | --- | --- |
| Anemia | 3 | Oncology consultation | € 78.84 | € 935.84 |  |
|  |  | Blood transfusion | € 857.00 |  |  |
|  |  |  |  |  |  |
|  | 4 | Oncology consultation | € 78.84 | € 935.84 |  |
|  |  | Blood transfusion | € 857.00 |  |  |
|  |  |  |  |  |  |
|  |  |  |  |  |  |
| Cutaneous squamous cell carcinoma | 3 | Oncology consultation | € 78.84 | € 1,063.22 |  |
|  |  | Excision | € 984.38 |  |  |
|  |  |  |  |  |  |
| Diarrhea | 3 | Oncology consultation | € 78.84 | € 85.70 |  |
|  |  | Loperamide | € 6.86 |  | 2 mg four times daily for 3-4 days |
|  |  |  |  |  |  |
|  | 4 | Oncology consultation | € 78.84 | € 85.70 |  |
|  |  | Loperamide | € 6.86 |  | 2 mg four times daily for 3-4 days |
|  |  |  |  |  |  |
| Diarrhea (immune-related) | 3 | Oncology consultation | € 78.84 | € 85.80 |  |
|  |  | Prednisone | € 6.96 |  | 1 mg/kg, 0.5 mg/kg and 0.25 mg/kg each once daily for a week (tapered) |
|  |  |  |  |  |  |
|  |  |  |  |  |  |
|  |  |  |  |  |  |
|  |  |  |  |  |  |
| Dyspnea | 3 | Oncology consultation | € 78.84 | € 188.34 |  |
|  |  | Oxygen | € 109.50 |  |  |
|  |  |  |  |  |  |
|  | 4 | 100% inpatient treatment |  | € 0.00 |  |
|  |  |  |  |  |  |
| Elevated liver enzymes | 3 | Oncology consultation | € 78.84 | € 78.84 |  |
|  |  |  |  |  |  |
|  | 4 | Oncology consultation | € 78.84 | € 78.84 |  |
|  |  |  |  |  |  |
| Febrile neutropenia | 3 | Oncology consultation | € 78.84 | € 81.39 |  |
|  |  | Amoxicillin/clavulanic acid (Augmentin) | € 2.54 |  | 875/125 mg twice daily for 7 days |
|  |  |  |  |  |  |
|  | 4 | Oncology consultation | € 78.84 | € 81.39 |  |
|  |  | Amoxicillin/clavulanic acid (Augmentin) | € 2.54 |  | 875/125 mg twice daily for 7 days |
|  |  |  |  |  |  |
| Fever | 3 | Oncology consultation | € 78.84 | € 81.60 |  |
|  |  | Paracetemol | € 2.76 |  | 1 gr four times daily for 7 days |
|  |  |  |  |  |  |
|  |  |  |  |  |  |
| Headache | 3 | Oncology consultation | € 78.84 | € 81.70 |  |
|  |  | Paracetemol | € 2.86 |  | 4 gr daily for 7 days |
|  |  |  |  |  |  |
| Hypertension | 3 | Oncology consultation | € 78.84 | € 79.33 |  |
|  |  | Amlopidine | € 0.49 |  | 5 mg/day for 30 days |
|  |  |  |  |  |  |
| Hypophysitis | 3 | Oncology consultation | € 78.84 | € 465.03 |  |
|  |  | Brain MRI | € 253.28 |  |  |
|  |  | Prednisone | € 132.91 |  | 1 mg/kg per day for 30 days |
|  |  |  |  |  |  |
| Infection | 3 | Oncology consultation | € 78.84 | € 81.39 |  |
|  |  | Amoxicillin/clavulanic acid (Augmentin) | € 2.54 |  | 875/125 mg twice daily for 7 days |
|  |  |  |  |  |  |
|  | 4 | 100% inpatient treatment |  | € 0.00 |  |
|  |  |  |  |  |  |
| Neutropenia | 3 | Oncology consultation | € 78.84 | € 78.84 |  |
|  |  |  |  |  |  |
|  |  |  |  |  |  |
|  | 4 | Oncology consultation | € 78.84 | € 78.84 |  |
|  |  |  |  |  |  |
| Palmar plantar hyperkeratosis | 3 | Oncology consultation | € 78.84 | € 157.68 |  |
|  |  | Dermatologist consultation | € 78.84 |  |  |
|  |  |  |  |  |  |
| Peripheral neuropathy | 3 | Oncology consultation | € 78.84 | € 83.24 |  |
|  |  | Gabapentin | € 4.40 |  | 300 mg for day 1, 600 mg for day 2 and 900 mg/day for days 3-30 |
|  |  |  |  |  |  |
|  | 4 | Oncology consultation | € 78.84 | € 83.24 |  |
|  |  | Gabapentin | € 4.40 |  | 300 mg for day 1, 600 mg for day 2 and 900 mg/day for days 3-30 |
|  |  |  |  |  |  |
| Rash | 3 | Oncology consultation | € 78.84 | € 86.35 |  |
|  |  | Prednisone | € 1.03 |  | 20 mg once daily for 7 days |
|  |  | Clemastine (Tavegil) | € 3.34 |  | 5 mg once daily for 7 days |
|  |  | Clobetasol (Dermovate) | € 3.14 |  | One tube |
|  |  |  |  |  |  |
|  | 4 | Oncology consultation | € 78.84 | € 81.98 |  |
|  |  | Clobetasol (Dermovate) | € 3.14 |  | One tube |
|  |  |  |  |  |  |
| Vomiting | 3 | Oncology consultation | € 78.84 | € 79.55 |  |
|  |  | Metoclopramide | € 0.71 |  | 20 mg/day for 5 days |
|  |  |  |  |  |  |
|  |  |  |  |  |  |
|  | 4 | Oncology consultation | € 78.84 | € 79.55 |  |
|  |  | Metoclopramide | € 0.71 |  | 20 mg/day for 5 days |

Table 6: Resource Use and Costs for the UK

| **Toxicity** | **Grade** | **Resource** | **Total resource cost** | **Total resource cost per toxicity** | **Comments** |
| --- | --- | --- | --- | --- | --- |
| Anemia | 3 | Oncology consultation | £125.68 | £729.78 |  |
|  |  | Admit for day case (transfusion) | £604.10 |  |  |
|  |  |  |  |  |  |
|  | 4 | Oncology consultation | £125.68 | £729.78 |  |
|  |  | Admit for day case (transfusion) | £604.10 |  |  |
|  |  |  |  |  |  |
| Cutaneous squamous cell carcinoma | 3 | Oncology consultation | £125.68 | £720.33 |  |
|  |  | Dermatology consultation | £106.43 |  |  |
|  |  | Surgery or radiotherapy | £488.21 |  |  |
|  |  |  |  |  |  |
| Diarrhea | 3 | 2 Oncology consultations | £251.37 | £251.37 |  |
|  |  |  |  |  |  |
|  | 4 | Oncology consultation | £125.68 | £125.68 |  |
|  |  |  |  |  |  |
| Diarrhea (immune-related) | 3 | 2 Oncology consultations | £251.37 | £251.37 |  |
|  |  |  |  |  |  |
| Dyspnea | 3 | 2 Oncology consultations | £251.37 | £251.37 |  |
|  |  |  |  |  |  |
|  | 4 | 100% inpatient admission |  | £0.00 |  |
|  |  |  |  |  |  |
| Elevated liver enzymes | 3 | 2 Oncology consultations | £251.37 | £251.37 |  |
|  |  |  |  |  |  |
|  | 4 | 2 Oncology consultations | £251.37 | £251.37 |  |
|  |  |  |  |  |  |
| Febrile neutropenia | 3 | 100% inpatient admission |  | £0.00 |  |
|  |  |  |  |  |  |
|  | 4 | 100% inpatient admission |  | £0.00 |  |
|  |  |  |  |  |  |
| Fever | 3 | 2 Oncology consultations | £251.37 | £251.37 |  |
|  |  |  |  |  |  |
| Headache | 3 | 2 Oncology consultations | £251.37 | £251.37 |  |
|  |  |  |  |  |  |
| Hypertension | 3 | 2 Oncology consultations | £251.37 | £251.37 |  |
|  |  | Anti-hypertensives^a^ |  |  |  |
|  |  |  |  |  |  |
|  |  |  |  |  |  |
|  |  |  |  |  |  |
| Hypophysitis | 3 | 2 Oncology consultations | £251.37 | £251.37 |  |
|  |  |  |  |  |  |
| Infection | 3 | 2 Oncology consultations | £251.37 | £251.37 |  |
|  |  |  |  |  |  |
|  | 4 | 2 Oncology consultations | £251.37 | £251.37 |  |
|  |  |  |  |  |  |
| Neutropenia | 3 | 2 Oncology consultations | £251.37 | £251.37 |  |
|  |  |  |  |  |  |
|  | 4 | 100% inpatient admission |  | £0.00 |  |
|  |  |  |  |  |  |
| Palmar plantar hyperkeratosis | 3 | Oncology consultation | £125.68 | £125.68 |  |
|  |  |  |  |  |  |
| Peripheral neuropathy | 3 | Oncology consultation | £125.68 | £125.68 |  |
|  |  |  |  |  |  |
|  | 4 | 2 Oncology consultations | £251.37 | £251.37 |  |
|  |  |  |  |  |  |
| Rash | 3 | 2 Oncology consultations | £251.37 | £251.37 |  |
|  |  | Diprobase^a^ |  |  |  |
|  |  | Topical corticosteroid^a^ |  |  |  |
|  |  | Anti-histamines^a^ |  |  |  |
|  |  |  |  |  |  |
|  | 4 | 2 Oncology consultations | £251.37 | £251.37 |  |
|  |  | Diprobase* |  |  |  |
|  |  | Topical corticosteroid^a^ |  |  |  |
|  |  | Anti-histamines^a^ |  |  |  |
|  |  |  |  |  |  |
| Vomiting | 3 | 2 Oncology consultations | £251.37 | £251.37 |  |
|  |  |  |  |  |  |
|  | 4 | 2 Oncology consultations | £251.37 | £251.37 |  |

^a^All drugs and tests, except GCSF, are included in the cost for an oncology consultation

Table 7: Resource Use and Costs for Australia

| **Toxicity** | **Grade** | **Resource** | **Total resource cost** | **Total resource cost per toxicity** | **Comments** |
| --- | --- | --- | --- | --- | --- |
| Anemia | 3 | Oncology consultation | $88.13 | $889.83 |  |
|  |  | Blood transfusion | $801.70 |  |  |
|  |  |  |  |  |  |
|  | 4 | Oncology consultation | $88.13 | $889.83 |  |
|  |  | Blood transfusion | $801.70 |  |  |
|  |  |  |  |  |  |
| Cutaneous squamous cell carcinoma | 3 | Oncology consultation | $88.13 | $423.74 |  |
|  |  | Dermatology consultation | $88.13 |  |  |
|  |  | Excision | $247.48 |  |  |
|  |  |  |  |  |  |
| Diarrhea | 3 | Oncology consultation | $88.13 | $90.60 |  |
|  |  | Imodium | $2.47 |  | 2 mg once daily for 3-4 days |
|  |  |  |  |  |  |
|  | 4 | Oncology consultation | $88.13 | $90.60 |  |
|  |  | Imodium | $2.47 |  | 2 mg once daily for 3-4 days |
|  |  |  |  |  |  |
| Diarrhea (immune-related) | 3 | Oncology consultation | $88.13 | $1,120.84 |  |
|  |  | 0.3 Infliximab | $1,019.19 |  | 5 mg/kg once |
|  |  | Prednisone | $13.51 |  | 1 mg/kg one week, 0.5 mg/kg one week, 0.25 mg/kg one week |
|  |  |  |  |  |  |
|  |  |  |  |  |  |
| Dyspnea | 3 | Oncology consultation | $88.13 | $128.84 |  |
|  |  | Oxygen | $0.00 |  |  |
|  |  | Dexamethasone | $4.29 |  | 4 mg twice daily for 5 days |
|  |  | Chest x-ray | $36.42 |  |  |
|  |  |  |  |  |  |
|  | 4 | 4 Oncology consultations | $352.52 | $393.23 |  |
|  |  | Oxygen | $0.00 |  |  |
|  |  | Dexamethasone | $4.29 |  | 4 mg twice daily for 5 days |
|  |  | Chest x-ray | $36.42 |  |  |
|  |  |  |  |  |  |
| Elevated liver enzymes | 3 | 2 Oncology consultations | $176.26 | $303.90 |  |
|  |  | 7 Liver function tests | $127.64 |  |  |
|  |  |  |  |  |  |
|  | 4 | 2 Oncology consultations | $176.26 | $303.90 |  |
|  |  | 7 Liver function tests | $127.64 |  |  |
|  |  |  |  |  |  |
| Febrile neutropenia | 3 | 100% inpatient treatment |  |  |  |
|  |  |  |  |  |  |
|  | 4 | 100% inpatient treatment |  |  |  |
|  |  |  |  |  |  |
| Fever | 3 | Oncology consultation | $88.13 | $93.57 |  |
|  |  | Paracetamol | $2.43 |  | 4 gr two times daily for 5 days |
|  |  | Dexamethasone | $3.00 |  | 4 mg once daily for 7 days |
|  |  |  |  |  |  |
| Headache | 3 | Oncology consultation | $88.13 | $98.93 |  |
|  |  | Paracetamol | $10.81 |  | 4 gr twice daily for 5 days |
|  |  |  |  |  |  |
|  |  |  |  |  |  |
| Hypertension | 3 | Oncology consultation | $88.13 | $97.25 |  |
|  |  | Amlodipine | $9.12 |  | 7.5 gr once daily for 30 days |
|  |  |  |  |  |  |
| Hypophysitis | 3 | Oncology consultation | $88.13 | $283.42 |  |
|  |  | 2 Endocrinologist consultations | $176.26 |  |  |
|  |  | Prednisone | $7.87 |  | 40 mg once daily for 14 days |
|  |  | Thyroid hormone replacement | $11.16 |  | 300 mcg once daily for 30 days |
|  |  |  |  |  |  |
| Infection | 3 | 100% inpatient treatment |  | $0.00 |  |
|  |  |  |  |  |  |
|  | 4 | 100% inpatient treatment |  | $0.00 |  |
|  |  |  |  |  |  |
| Neutropenia | 3 | Oncology consultation | $88.13 | $140.51 |  |
|  |  | 3 Blood tests | $52.38 |  |  |
|  |  |  |  |  |  |
|  | 4 | Oncology consultation | $88.13 | $210.36 |  |
|  |  | 7 Blood tests | $122.23 |  |  |
|  |  |  |  |  |  |
| Palmar plantar hyperkeratosis | 3 | Oncology consultation | $88.13 | $203.47 |  |
|  |  | Dermatologist consultation | $88.13 |  |  |
|  |  | 10% urea cream | $12.66 |  | One tube |
|  |  | Salicylate cream | $14.55 |  | One tube |
|  |  |  |  |  |  |
| Peripheral neuropathy | 3 | Oncology consultation | $88.13 | $152.26 |  |
|  |  | Podiatry consultation | $64.13 |  |  |
|  |  |  |  |  |  |
|  | 4 | Oncology consultation | $88.13 | $218.06 |  |
|  |  | Podiatry consultation | $64.13 |  |  |
|  |  | 0.5 Physio, Occupational therapist | $44.06 |  |  |
|  |  |  |  |  |  |
| Rash | 3 | 4 Oncology consultation | $352.52 | $379.68 |  |
|  |  | Clobetasol cream | $8.75 |  | One tube |
|  |  | Prednisone | $11.59 |  | Starting dose of 50 mg daily for 3 days, tapered by 5 mg every 3 days |
|  |  | Loratadine | $6.82 |  | 10 mg once daily for 6 days |
|  |  |  |  |  |  |
|  | 4 | 3 Oncology consultation | $264.39 | $372.86 |  |
|  |  | Dermatology consultation | $88.13 |  |  |
|  |  | Clobetasol cream | $8.75 |  | One tube |
|  |  | Prednisone | $11.59 |  | Starting dose of 50 mg daily for 3 days, tapered by 5 mg every 3 days |
|  |  |  |  |  |  |
|  |  |  |  |  |  |
| Vomiting | 3 | Oncology consultation | $88.13 | $146.84 |  |
|  |  | Ondansentron (Zofran) | $58.71 |  | 8 mg twice daily for 3-4 days |
|  |  |  |  |  |  |
|  | 4 | 100% inpatient admission |  | $0.00 |  |

Table 8: Resource Use and Costs for Canada

| **Toxicity** | **Grade** | **Resource** | **Total resource cost** | **Total resource cost per toxicity** | **Comments** |
| --- | --- | --- | --- | --- | --- |
| Anemia | 3 | Oncology consultation | $160.45 | $370.41 |  |
|  |  | Blood transfusion | $209.96 |  |  |
|  |  |  |  |  |  |
|  | 4 | Oncology consultation | $160.45 | $370.41 |  |
|  |  | Blood transfusion | $209.96 |  |  |
|  |  |  |  |  |  |
| Cutaneous squamous cell carcinoma | 3 | Oncology consultation | $160.45 | $204.55 |  |
|  |  | Excision | $44.10 |  |  |
|  |  |  |  |  |  |
| Diarrhea | 3 | Oncology consultation | $160.45 | $161.81 |  |
|  |  | Imodium | $1.36 |  | 2 mg four times daily for 3-4 days |
|  |  |  |  |  |  |
|  | 4 | Oncology consultation | $160.45 | $161.81 |  |
|  |  | Imodium | $1.36 |  | 2 mg four times daily for 3-4 days |
|  |  |  |  |  |  |
| Diarrhea (immune-related) | 3 | Oncology consultation | $160.45 | $161.74 |  |
|  |  | Prednisone | $1.29 |  | Initial dose of 40 mg/day, with a weekly taper of 10 mg/day for a total of 4 weeks of treatment |
|  |  |  |  |  |  |
|  |  |  |  |  |  |
| Dyspnea | 3 | Oncology consultation | $160.45 | $193.82 |  |
|  |  | Chest x-ray | $33.37 |  |  |
|  |  |  |  |  |  |
|  | 4 | Oncology consultation | $160.45 | $193.82 |  |
|  |  | Chest x-ray | $33.37 |  |  |
|  |  |  |  |  |  |
|  |  |  |  |  |  |
| Elevated liver enzymes | 3 | Oncology consultation | $160.45 | $160.45 |  |
|  |  |  |  |  |  |
|  | 4 | Oncology consultation | $160.45 | $160.45 |  |
|  |  |  |  |  |  |
| Febrile neutropenia | 3 | Oncology consultation | $160.45 | $257.57 |  |
|  |  | Blood count | $16.38 |  |  |
|  |  | Gentamicin | $80.73 |  | 1 mg/kg three times daily for 7 days |
|  |  |  |  |  |  |
|  | 4 | Oncology consultation | $160.45 | $257.57 |  |
|  |  | Blood count | $16.38 |  |  |
|  |  | Gentamicin | $80.73 |  | 1 mg/kg three times daily for 7 days |
|  |  |  |  |  |  |
| Fever | 3 | Oncology consultation | $160.45 | $161.27 |  |
|  |  | Paracetamol | $0.82 |  | 1 gram four times daily for 1 week |
|  |  |  |  |  |  |
| Headache | 3 | Oncology consultation | $160.45 | $226.83 |  |
|  |  | Brain CT | $66.38 |  |  |
|  |  |  |  |  |  |
|  |  |  |  |  |  |
|  |  |  |  |  |  |
| Hypertension | 3 | Oncology consultation | $160.45 | $164.28 |  |
|  |  | Metoprolol | $3.83 |  | 50 mg twice daily for 30 days |
|  |  |  |  |  |  |
| Hypophysitis | 3 | Oncology consultation | $160.45 | $168.40 |  |
|  |  | Prednisone | $4.25 |  | 1 mg/kg daily for 30 days |
|  |  | Thyroid hormone replacement | $3.69 |  | 125 mcg/day for 30 days |
|  |  |  |  |  |  |
| Infection | 3 | Oncology consultation | $160.45 | $161.43 |  |
|  |  | Sulfamethoxazole/Trimethoprim (Septra) | $0.99 |  | 80 mg twice daily for 10 days |
|  |  |  |  |  |  |
|  |  |  |  |  |  |
|  | 4 | Oncology consultation | $160.45 | $161.43 |  |
|  |  | Sulfamethoxazole/Trimethoprim (Septra) | $0.99 |  | 80 mg twice daily for 10 days |
|  |  |  |  |  |  |
| Neutropenia | 3 | Oncology consultation | $160.45 | $160.45 |  |
|  |  |  |  |  |  |
|  | 4 | Oncology consultation | $160.45 | $160.45 |  |
|  |  |  |  |  |  |
| Palmar plantar hyperkeratosis | 3 | Oncology consultation | $160.45 | $160.45 |  |
|  |  | Moisturizer | $0.00 |  |  |
|  |  |  |  |  |  |
|  |  |  |  |  |  |
| Peripheral neuropathy | 3 | Oncology consultation | $160.45 | $183.37 |  |
|  |  | Gabapentin | $22.92 |  | 300 mg on day 1, 600 mg on day 2, 900 mg on days 3-30 |
|  |  |  |  |  |  |
|  |  |  |  |  |  |
|  |  |  |  |  |  |
|  |  |  |  |  |  |
|  | 4 | Oncology consultation | $160.45 | $210.40 |  |
|  |  | Gabapentin | $22.92 |  | 300 mg on day 1, 600 mg on day 2, 900 mg on days 3-30 |
|  |  | 0.15 Neurology consultation | $27.03 |  |  |
|  |  |  |  |  |  |
|  |  |  |  |  |  |
|  |  |  |  |  |  |
|  |  |  |  |  |  |
| Rash | 3 | Oncology consultation | $160.45 | $170.91 |  |
|  |  | Betamethasone cream | $10.46 |  | One tube |
|  |  |  |  |  |  |
|  |  |  |  |  |  |
|  |  |  |  |  |  |
|  | 4 | Oncology consultation | $160.45 | $161.76 |  |
|  |  | Prednisone | $1.31 |  | Initial dose of 40 mg/day, with a weekly taper of 10 mg/day for a total of 4 weeks of treatment |
|  |  |  |  |  |  |
|  |  |  |  |  |  |
|  |  |  |  |  |  |
| Vomiting | 3 | Oncology consultation | $160.45 | $239.44 |  |
|  |  | Ondansetron (Zofran) | $78.99 |  | 8 mg twice daily for 2 days |
|  |  |  |  |  |  |
|  |  |  |  |  |  |
|  | 4 | Oncology consultation | $160.45 | $239.44 |  |
|  |  | Ondansetron (Zofran) | $78.99 |  | 8 mg twice daily for 2 days |
